# Integration in health: cooperation at triple international border Amazon

**DOI:** 10.11606/s1518-8787.2020054001306

**Published:** 2019-12-23

**Authors:** Giane Zupellari dos Santos-Melo, Selma Regina de Andrade, Betina Hörner Schlindwein Meirelles, Angela Maria Blatt Ortiga

**Affiliations:** I Universidade do Estado do Amazonas. Departamento de Enfermagem. Manaus, Amazonas, Brasil.; II Universidade Federal de Santa Catarina. Departamento de Pós-Graduação em Enfermagem. Florianópolis, Santa Catarina, Brasil.; III Governo do Estado de Santa Catarina. Secretaria de Estado da Saúde de Santa Catarina. Florianópolis, Santa Catarina, Brasil.

**Keywords:** International Cooperation, Health Services Administration, Program Evaluation, Border Areas, Border Health

## Abstract

**OBJECTIVE:**

To describe the scope and limitations of the main strategies of cooperation in health, adopted between 2005 and 2017, in the context of the triple border Brazil, Colombia and Peru.

**METHOD:**

Single, explanatory, qualitative, integrated case study carried out in 2017, in the context of the triple Amazon border, Brazil, Colombia and Peru, in the city of Tabatinga, state of Amazonas, Brazil. Our sources of evidence were: documentary data; interviews with health managers of the State Health Secretariats of Amazonas and Municipal Health of Tabatinga, Municipal Health Council of Tabatinga and Consulate of Peru in Colombia; and direct observations in four health services of Tabatinga. Data were organized with MaxQDA12^®^ software.

**RESULTS:**

Data analyzed showed that, during the study period, the Brazilian federal government made several health cooperation agreements with both Peru and Colombia and that the state government of Amazonas undertook strategies to improve the health conditions of the dwellers of Tabatinga and the region of Alto Solimões, which indirectly reached the populations of neighboring countries, supporting the interrelationships between the countries of the region. Regarding the municipal government, we verified the existence of health integration agreements, established informally, to minimize the adversities of the local health.

**CONCLUSION:**

The cooperation strategies in health adopted in the triple Amazon border have different purposes, benefits and limitations. It is noteworthy that the existence of cooperation agreements between the federal governments of Brazil, Colombia and Peru and the presence of informal cooperation agreements between the municipal governments of Tabatinga (Brazil), Leticia (Colombia) and Santa Rosa (Peru). The limitations of this study are the lack of knowledge of local managers about the cooperation agreements established between federal governments and the lack of legitimacy of the informal agreements established by the Tabatinga government.

## INTRODUCTION

Nowadays, Brazil maintains diplomatic relations with South American countries and health institutions. Recently, international organizations of which Brazil is a member have provided an evolution in the cooperation treaty with South America, enabling thus an area of integration in health among countries^1^^,^^2^.

In border regions, these relationships tend to intensify, since they are considered preliminary steps for regional integration processes^3^. It is noteworthy that the cooperation between health systems, recognized as one of the main initiatives to support these approches^4^.

Integration in health in international border regions are procedures for convergence, approximation and harmonization of policies, regulations and actions that provide access to common institutions, with consequent permission to consumption of social services among countries^4^^,^^5^. With this understanding, the cooperation addressed in this study comprises strategies that support, to a greater or lesser extent, the integration in health at its various aspects, from the collaboration between local health systems to the generation of knowledge in the area^4^.

In global terms, the defense that health actions and knowledge distribution in the area cannot be restricted to a particular country is emphatic, for it is a public good that should be available to all people and regions^6^^,^^7^. Therefore, there is the recognition that integration in health among countries tends to strengthen equity at world levels^6^^,^^7^.

The border town of Tabatinga, which served as case in this study, integrates the Legal Amazon, also known as the “triple Amazon border,” because it forms a sister city with Leticia, the capital of the Amazonas Department, Colombia, as well as a river boundary with the City of Santa Rosa do Yavarí, Department of Loreto, Peru.

The socioeconomic, environmental and cultural conditions of the populations in the triple Amazon border are portrayed as precarious due to low income, deforestation, and illegal drug trade existing in the region^8^. In addition to these characteristics, the three countries present contrasting health systems, which complicates the integration-in-health processes of the region^9^, with Brazil being the only one structured under the principle of universality^10^.

To study how the processes of integration in health are defined between the countries of the triple Amazon border can strengthen the discussions on this topic and elucidate how such mechanisms can be conducted to improve the health needs in border regions. Thus, this study aims to describe the scope and limitations of the main strategies of health cooperation, adopted between 2005 and 2017, in the context of the triple border Brazil, Colombia and Peru.

## METHODS

This is a single, explanatory, qualitative and integrated case study^11^, carried out in the context of the triple border Brazil, Colombia and Peru, taking as case the municipality of Tabatinga, Amazonas, Brazil. The municipality has 63,635 dwellers, and it is located in the northern region of the country, situated on the left bank of the Solimões River, in the microregion of Alto Solimões, 1,105 km from the state capital^12^. Tabatinga was chosen for being at the border with the municipality of Leticia, far south of the Amazonas Department, Colombia, and with the island of Santa Rosa do Yavarí, located on the Maranon River (natural border), Mariscal Ramón Castilla Province, Department of Loreto, Peru.

To ensure the study quality, reliability and validity tests were employed^11^. This study used documentary data, interviews, and direct observations^11^ as sources of evidence, which allowed a wide approach of the studied phenomenon^13^.

The collection of documentary data^11^ occurred in March 2017, in physical archives of the Municipal Health Secretariat and the Municipal Health Council of Tabatinga, as well as in websites of the State Secretariat of Health of Amazonas and the Brazilian Ministry of Health. The study included administrative acts, decrees, laws, ordinances and resolutions issued by the Ministry of Health; minutes and resolutions issued by the Bipartite Inter-managers Commission of the State of Amazonas and the Regional Inter-managers Commission of Alto Solimões; and official correspondences, resolutions and minutes issued by the Municipal Health Secretariat and by the Municipal Health Council of Tabatinga, all produced between 2005 and 2017. These documents were chosen due to the authenticity of information about possible processes of integration in health on border areas as well as in the triple border. The temporary cut was considered based on the implementation of the *Sistema Integrado de Saúde das Fronteiras* (SIS Fronteiras – Integrated Border Health System) in Brazil^14^. The documents were assessed according to their structure and content, related to the proposed theme, generating a database of 98 documents.

The “short case study interviews”^11^ occurred between April and November 2017. In total, 12 interviews were conducted with managers of the state and municipal health departments and the Municipal Health Council (Table 1). The participants were selected for presenting decision-making power related to the processes of integration in health between the countries of the triple border. A key informant from the Consulate of Peru in Colombia was interviewed, which was fundamental to understand the phenomenon, as he presented strong evidence, representing one of the foreign countries that compose the triple Amazon border. The meetings were previously scheduled with the managers and counted with a semi-structured interview script, containing a question directed to the understanding of the phenomenon: “Do you know about the existence of formal or informal strategies or agreements that contribute to the assistance of the integration in health between Tabatinga (Brazil), Leticia (Colombia) and Santa Rosa Island (Peru)?” The interviews lasted 60 minutes and were conducted and transcribed by one of the researchers.

**Table 1:** Participants in the case study on cross-border health integration in the triple international border of Amazonia, Brazil.

State Manager	Secretary of the health condition of Amazonas; Coordinator of the Executive Secretariat of Specialized Healthcare of the Interior of the State Secretariat of Health of Amazonas; Coordinator of the Regional Inter-managers Commission of Alto Solimões; Coordinator of the Urgent Care Center of Tabatinga; Director of the Maternity Celina Villacrez Ruiz de Tabatinga; Director of the Health Surveillance Foundation of the state of Amazonas.
Municipal Manager	Secretary of the Municipal Health of Tabatinga; President of the Municipal Health Council of Tabatinga; Coordinator of primary care in the municipality of Tabatinga; Coordinator of the Dídimo Pires de Oliveira basic family health unit; Coordinator of the Santa Rosa basic family health unit .
Key informant	Consul of Peru in Colombia.

The direct observation^11^ occurred in the months of November and December 2017, generating a total of 34 observation hours. Two Basic Family Health Units (BFHU) were included in the study, selected as observation points for being situated in the vicinity of the international border lines. These two BFHU are administered by the Municipal Health Secretariat and are located in the neighborhoods of São Francisco and Santa Rosa.

Other services observed were an urgent care center and a state maternity, chosen as an observation point for being references for urgency and emergency care and low-risk deliveries in the Alto Solimões region, in addition to providing health care to foreigners.

Both units are managed by the State Secretariat, but located in the Vila Paraíso neighborhood, in Tabatinga.

To guide the observations, a script was used, composed of: place, date and time of beginning and ending of observations; observed events; and actions or situations that demonstrate the existence or necessity of the existence of integration agreements in health between the countries of the triple border.

Data were organized with the aid of MAXQDA12^®^ software, which enabled the creation of codes and subcodes^11^. The confrontation of the three evidence sources enabled data triangulation concerning the existence of informal agreements related to the results presented (Figure 1).

**Figure:**
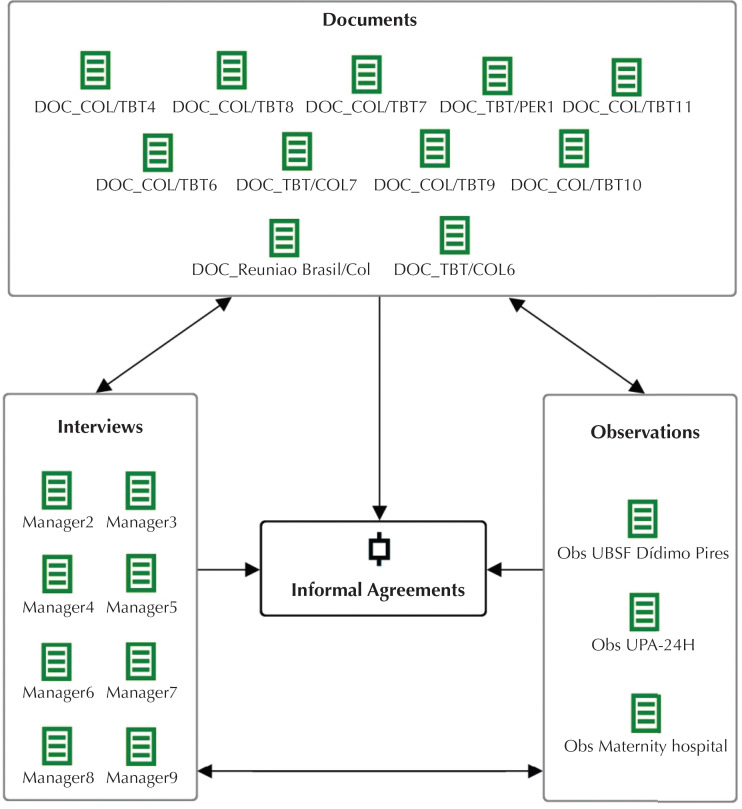
Triangulation of data evidencing the existence of informal agreements of integration in health in the Amazonian triple border Brazil, Colombia and Peru.

Source: MaxQDA12^®^, 2018.

Obs: Observation

The ethical guidelines of the National Health Council were respected in all phases of the study, which received the approval by the Research Ethics Committee of the Universidade Federal de Santa Catarina, with Opinion No. 2.047.137.

## RESULTS AND DISCUSSION

We evidenced that in the study period the governments of Brazil, Colombia, and Peru conducted several health cooperation agreements, as shown in Table 2. Although these agreements remain operative, we verified that the participants of this study ignore their existence or do not recognize them as possible support tools to the integration in health in the region.

**Table 2:** Agreements of integration in health between Brazil and Colombia or Brazil and Peru, from 2005 to 2017.

Year*	Countries	Agreement goal	Validity
2006	Brazil and Peru	Strengthening of regulation and inspection of public health in the decentralization process of Ministries of Health in Brazil and Peru.	Operative
2006	Brazil and Peru	Institutional strengthening of the international advisors of the Ministries of Health in Brazil and Peru.	Operative
2007	Brazil and Colombia	Technical support for the implementation of human milk banks in Colombia.	Operative
2007	Brazil and Colombia	Institutional strengthening of the international advisors of the Brazilian and Colombian Ministries of Health.	Operative
2009	Brazil and Colombia	Strengthening in the molecular diagnosis and typification of the species of *Leishmania*, its georeferencing and spatial analysis.	Operative
2010	Brazil and Peru	Establishment of an integrative border zone Brazil-Peru for the creation of the subgroup of work on health at the border.	Operative
2012	Brazil and Colombia	Research and development for the manufacture and control of the organic products quality in Colombia.	Operative
2013	Brazil and Peru	Establishment of a complementary framework to develop a new cooperative program between the Brazilian Ministry of Health as well as associated organizations and the Peruvian Ministry of Health.	Operative

* Year of the Agreement ratification.

Formal agreement involving the triple border? I've never heard of it. […] Only if they are within the region itself, […] But to cover the municipalities of neighboring countries, no – that is, these international issues. (Manager 8)

I've heard some bilateral agreements have been signed, but I know that in reality, here, these agreements have never worked. […] I know they exist, but they don't work. (Manager 6)

The agreements concluded between the federal governments of Brazil, Colombia and Peru, follow the global order, which increasingly understands that international cooperation is an expected initiative among countries, believing that the exchange of knowledge and experiences can level the nations^15^. However, for this cooperation to present effective results, it is necessary to create norms, mobilize resources and guide the various parties involved^16^, which in border regions are dwellers, managers and health professionals^10^.

This study collected data on two strategies, established by the federal government, with the capacity to promote integration in health in the study region. The first strategy was the SIS Fronteiras, created in 2005 to assess the health conditions on border municipalities and promote the integration of health actions and services. The municipality of Tabatinga adhered to the program in 2006, but the data analyzed showed that, in the region of the triple Amazon border, the SIS Fronteiras supported few advances.

The matter of integration, I don't know how the SIS Fronteiras intended to do this, because here in the region, nothing happened in relation to this proposal. (Manager 6)

The second strategy was the *Plano de Desenvolvimento e Integração das Faixas de Fronteira* (PDIFF – Development and Integration Plan of the Frontier Lines), which aims to promote the development of the border by physical, social and productive structuring. This strategy dialogued with managers of the Triple border Brazil, Colombia and Peru. However, this dialogue did not increase expectations and credibility in possible changes in policies aimed at integrating health.

I attended one of these meetings in Brasilia […] I spoke about the health difficulties, […] But the deal is: for the meeting they made themselves available, but it's been sometime since I came back from there and no one else has said anything. (Manager 6)

Although these two strategies aim to support integration in health on border regions, the participants do not recognize their effectiveness in the study region. In the SIS Fronteiras case, this may be related to distant and small municipalities, such as the one studied, the implementation program was made vertically, with a reduced debate between the ministerial, state and municipal bodies, leading to unawareness and misinterpretations of the program and its results^17^. For these municipalities, the conception of the SIS Fronteiras was linked to the financial funding for the health care of foreigners, being little associated with cross-border cooperation.

Regarding the government of Amazonas, one can verify that it undertook strategies to improve the health conditions of the local population, which indirectly reached the dwellers of neighboring countries seeking health care in Tabatinga, benefiting thus the interrelationship between the three countries. However, we observed these strategies were not specifically formulated to support the integration in health in the triple border, nor to create rules for dwellers' health care in the neighboring countries of Brazil, so the practice of health care abroad remains a discretion of the local manager.

By recognizing the peculiarities of the border regions that constitute its territory, the state government undertakes such strategies, by verticalized policies of the federal government, to provide answers to the health needs of the Brazilian population in that region. However, even admitting the existence of difficulties related to the health care of foreigners, the state government does not promote direct actions that support the integration of health, for it lacks political-administrative autonomy to establish International agreements with neighboring countries. These non-central institutions such as states and municipalities may establish formal cooperation agreements with international governments, since they have an approval of the central power as well as the seal of the Federal Senate, but such agreements have legal fragility, since they do not present legal instruments that sustain them^18^.

In this context, the low autonomy of local governments results in difficulties in the coalition of measures to improve the health of frontier populations. Regardless of whether the Federal Constitution raises states to federative entities, this does not legitimize that they autonomously establish executions with international organizations, becoming thus dependent on guidelines proposed by the federal government^19^. This dependence is generated by the vertical model of social policy implementation — including health — of the Brazilian government^20^, since institutional mechanisms such as the concentration of tax resources in the federal government limit the decision-making autonomy of local governments^21^. Thus, for policies of this nature to be effective, they must be planned horizontally, respecting the directives of intersectoriality, relations among federal, state and municipal governments and the political and territorial dimensions^20^.

In the case of border regions, the pact for health has brought advances by covering the frontier territories in its regionalization project. In addition to ensuring the compliance with the principles of universality, equity and completeness of health actions and services, this determination expanded the operational capacity of municipalities. However, the implementation of this norm depends on the possibility of these municipalities to exercise their autonomy in health management^22^.

Among the strategies implemented by the state government of Amazonas, three focused on health conditions on the triple border. One of them was the *Projeto de Desenvolvimento Regional do Estado do Amazonas* (Proderam — Regional Development Project of the state of Amazonas), implemented in Tabatinga in 2003, with the objective of improving the quality of life of the local population by assuring the access to health, improving the basic sanitation systems, and increasing job vacancies as well as the family income. Another strategy was the *Projeto de Formação e Melhoria da Qualidade da Rede de Saúde* (QualiSUS-Rede — Project of Training and Improvement of the Health Network Quality), which aimed to organize regionalized health care networks in Brazil. This program has advanced in the region from 2012 onwards.

These two strategies were essential for the creation of the *Rede de Atenção à Saúde do Alto Solimões* (RAS-AS – Alto Solimões Health Care Network), of which Tabatinga is a polo municipality. With the experience of the Proderam, the Alto Solimões region was chosen as one of the 15 health regions to participate in the QualiSUS-Rede in the Amazon region. RAS-AS provided to Tabatinga the infrastructure to urgency and emergency care as well as to women's and children's health. These strategies changed the characteristics of the region, including the health care of foreigners, who began to rely on these services.

Usually, they come for emergency situations […] The Peruvian dwellers sometimes receive care at Tabatinga […] In the urgent care center (UCC). (Manager 11)

The Pact for Health, in the dimension of the Management Pact, determines the design of regionalized health care networks in the Brazilian Unified Health System (SUS). This proposal has evolved into the health care networks, which are nowadays one of the main proposals for the functioning of health services^23^. The implementation of RAS-AS was a strategy, in which there was a slight alteration in the interrelationship between federal and state governments, as health and geography conditions in the triple border were considered priority for the implantation of a health care network in that region^24^^,^^25^.

The RAS-AS is understood by most of the participants as being able to reduce the social inequalities of the region. However, some argue that, despite improving the resolutiveness of the health sector, it does not contemplate the magnitude of the local reality. For them, the answers are imposed by normative instructions that usually do not correspond to the needs of the frontier population.

Health tries to answers based on the general views, by primary care assistance, urgency and emergency, hospitalizations, delivery and birth, […] but we know that this doesn't reach their needs. (Manager 7)

Thus, in order to recognize the real needs of the region, the state government has developed the strategy of maintaining institutional supporters in RAS-AS. Their function is to observe the health dynamics and suggest actions that can minimize the social inequalities found.

Our vision today is by region. The state will intervene in the triple border by institutional supporters and the situational diagnosis of the region. […] This institutional supporter will monitor the municipalities of the region to understand and give a better deal to local specificities. (Manager 7)

The conception of institutional support in the health sector is a management mechanism based on the Paideia method, which transforms the way to perform health management. By co-management among managers, workers, and users, this method is able to expand the reflective analysis of the collective. Thus, the institutional supporter is considered a resource for the construction of changes in groups and health organizations^26^.

Regarding the strategies established by the municipal government to promote integration, we observed the occurrence of intense health cooperative agreements, carried out informally between Tabatinga and the two municipalities of neighboring countries. These agreements are mostly concluded between the Colombian and Brazilian municipalities and used both for information exchange and for health goods and services. One of the participants makes it clear that they are common in the region:

Today, we work with the integration, even if it's formally, but there is integration, […] here is kind of automatic, because we have to adapt to this phenomenon that is the frontier. And how do we do that? Making partnerships. (Manager 6):

These types of agreement, although not legally recognized^19^, are common among international border municipalities, both in Brazil and in other countries^27^. Nowadays, this phenomenon, described as paradiplomacy, has been gaining prominence in the discussions of international relations and redefining the role of foreign relations in the world, mainly in the Americas, Western Europe and Asia^28^.

Paradiplomacy can be defined as the participation of federative or regional entities in international affairs, such as the execution of treaties with foreign States, participation in international networks of regional cooperation and action in external policies, without the support of the central governments^29^.

In border regions, the phenomenon of paradiplomacy tends to intensify, for the mutual needs of the regions, usually marked by socioeconomic and environmental imbalances, as well as geographical characteristics, make governments and non-governmental institutions unite to minimize regional iniquities^27^. Therefore, this type of cross-border cooperation enables local governments with scarce resources and low presence of the nation states to find objective, fast and creative solutions to the regional problems^29^.

Paradiplomacy is used by the health managers of the triple Amazon border as a strategy to avoid the adversities of the region, especially those related to logistics, which is hindered by the high distance between the municipality and the state capital, and to minimize the low resolutiveness of local health services. Some participants in this study stated that this tool has been fundamental in the development of health actions in the region.

The oxygen plant supply here doesn't meet our needs, […] So Leticia is the one who supplies me. […] Through informal agreements. (Manager 3)

If someday they need anything, and we have it, we will help, and if we need anything, if they can, they will help us. But it depends on the contacts. […] It's all gentlemen's agreement, you know, good neighborliness. (Manager 6)

By national laws, the Brazilian municipality of the triple Amazon border may conclude such agreements, provided that they are ratified by the central government^18^^,^^19^. The Colombian municipality of Leticia is supported by the Constitution of Colombia, which allows border municipalities to establish bi-national agreements for economic development and articulations with neighboring countries, provided they are geared towards the improvement of life conditions and social inclusion of isolated communities. Therefore, the Embassy of Colombia in Tabatinga enables the establishment of cooperation agreements between the two municipalities on topics related to education, public safety, transportation, and health^30^.

The differences in the legal treaties related to this topic in the two countries cause insecure situations for managers on the Brazilian side, which acknowledge that the existing agreements, nowadays, do not have legal validity and declare discomfort in promoting them. However, they affirm they constitute a local alternative to supply the lack of actions that favor the integration in health on the region by the higher levels.

All of this makes me very restless, because there is nothing formal in a higher sphere. (Manager 3)

Although national regulations indicate these kinds of agreements are not official, the Brazilian federal government follows guidelines for the paradiplomacy acceptability, which confers certain autonomy for Brazilian border municipalities to perform these transactions. The prerogative of the paradiplomacy acceptability in border regions is already recognized in countries such as Canada, Mexico, Germany, Argentina, South Africa, and the United States, because they are considered beneficial, given the political diversity they provide^19^^,^^20^^,^^31^.

## CONCLUSION

For being normally isolated territories with peculiar socioeconomic characteristics, international boundaries are recognized as favorable territories for the development of political, economic and cultural integration processes that tend to minimize the inequalities existing in these locations. This study aimed to clarify the occurrence of health cooperation strategies in the triple border region of Brazil, Colombia and Peru. We observed that these strategies exist, but present different purposes, depending on the policies involved and the governmental level that establishes them. The agreements signed have little effectiveness in the region, and the government of Amazonas does not promote specific strategies to support the integration in health.

Thus, there is a distance between municipality and other governmental levels, resulting in informal cooperation agreements between Tabatinga, Leticia and Santa Rosa Island. These agreements represent an alternative to minimize health inequities and could serve as precedents for future proposals to integrate higher levels of the governments of Brazil, Colombia and Peru. In agreement with the terms of global health, the involvement of federal and state governments in negotiations of this nature may favor the improvement of conditions in the region, making the access to health goods and services less exclusive and more equitable .

## References

[1] 1. Silva SAG, Duarte RG, Castro JM. Transfer of knowledge in international cooperation: the Farmanguinhos – SMM case. Rev Saude Publica. 2017;51:103. https://doi.olrg/10.11606/S1518-8787.2017051006249 10.11606/S1518-8787.2017051006249 PMC5697924 29166441

[2] 2. Faria M, Giovanella L, Bermudez L. A Unasul na Assembleia Mundial da Saúde: posicionamentos comuns do Conselho de Saúde Sul-Americano. Saude Debate. 2015;39(107):920-34. https://doi.org/10.1590/0103-110420151070230

[3] 3. Guimarães L, Giovanella L. Municípios brasileiros fronteiriços e MERCOSUL: características e iniciativas de cooperação em saúde. Saude Debate. 2005 [citado 15 ago 2018];29(71):248-57. Disponível em: http://www6.ensp.fiocruz.br/repositorio/sites/default/files/arquivos/Municipios.pdf

[4] 4. Bontempo CGC, Nogueira VMR, Gimenez RP. Cooperação em saúde em fronteiras internacionais: a busca da igualdade em saúde. Cad Ibero-Am Dir Sanit. 2013;2(2):908-19. https://doi.org/10.17566/ciads.v2i2.131

[5] 5. Guimarães L, Giovanella L. Integração européia e políticas de saúde: repercussões do mercado interno europeu no acesso aos serviços de saúde. Cad Saude Publica. 2006;22(9):1795-807. https://doi.org/10.1590/S0102-311X2006000900010 10.1590/s0102-311x2006000900010 16917576

[6] 6. Stewart KA, Keusch GT, Kleinman A. Values and moral experience in global health: bridging the local and the global. Global Public Health. 2010;5(2):115-21. https://doi.org/10.1080/17441690903484201 10.1080/17441690903484201 20213562

[7] 7. Feierman S, Kleinman A, Stewart K, Farmer D, Das V. Anthropology, knowledge-flows and global health. Global Public Health. 2010;5(2):122-8. https://doi.org/10.1080/17441690903401338 10.1080/17441690903401338 20013523

[8] 8. Padilla JD, Pimentel LC, Luján PM, Gomes DAL. Estrategias de ocupación del gobierno central en la Amazonia colombiana. Rev Geopol Transfront. 2017 [citado 16 ago 2018];1(2):60-80. Disponível em: http://periodicos.uea.edu.br/index.php/revistageotransfronteirica/article/view/780/674

[9] 9. Guerra K, Ventura M. Bioética, imigração e assistência à saúde: tensões e convergências sobre o direito humano à saúde no Brasil na integração regional dos países. Cad Saude Coletiva. 2017;25(1):123-9. https://doi.org/10.1590/1414-462X201700010185

[10] 10. Cárdenas WIL, Pereira AMM, Machado CV. Trajetória das relações público-privadas no sistema de saúde da Colômbia de 1991 a 2015. Cad Saude Publica. 2017;33(2). https://doi.org/10.1590/0102-311X00114016 10.1590/0102-311X00114016 28767814

[11] 11. Yin RK. Estudo de caso: planejamento e métodos. 5. ed. Porto Alegre: Bookman, 2015.

[12] 12. Instituto Brasileiro de Geografia e Estatística. Cidades: Tabatinga. Rio de Janeiro, RJ: IBGE; 2018 [citado 10 jun 2018. Disponível em: https://cidades.ibge.gov.br/brasil/am/tabatinga/panorama

[13] 13. Andrade SR, Ruoff AB, Piccoli T, Schmitt MD, Ferreira A, Xavier ACA. Case study as a nursing research method: an integrative review. Texto Contexto Enferm. 2017;26(4):e5360016. https://doi.org/10.1590/0104-07072017005360016

[14] 14. Ministério da Saúde (BR). Portaria Nº 1.120, de 6 de julho de 2005. Institui o Sistema Integrado de Saúde das Fronteiras - SIS FRONTEIRAS. Diario Oficial Uniao. 7 jul 2005; Seção 1:47.

[15] 15. Ferreira JR, Hoirisch C, Fonseca LE, Buss PM. Cooperação internacional em saúde: o caso da Fiocruz. Hist Cienc Saude Manguinhos. 2016;23(2):267-76. https://doi.org/10.1590/S0104-59702016000200002 10.1590/S0104-59702016000200002 27280315

[16] 16. Gostin LO, Sridhar D. Global health and the law. N Engl J Med. 2014;370(18):1732-40. https://doi.org/10.1056/NEJMra1314094 10.1056/NEJMra1314094 24785208

[17] 17. Nogueira VMR, Fagundes HS. A implementação do SIS fronteiras – perspectivas para a ampliação do direito à saúde na fronteira arco sul. Serv Soc Saude. 2014;13(2):245-60. https://doi.org/10.20396/sss.v13i2.8634903

[18] 18. Pereira JA, Luz CK. Fundamentos constitucionais e os projetos legislativos na paradiplomacia para pequenos e médios municípios: quando o global ainda mora longe do local. Campo Jurídico. 2017 [citado 16 jun 2018];5(1):161-9. Disponível em: http://www.fasb.edu.br/revista/index.php/campojuridico/article/view/185/184

[19] 19. Junqueira CGB. A criação das secretarias municipais de relações internacionais (SMRIS) como nova realidade da inserção internacional dos entes subnacionais brasileiros. Bol Econ Polit Int. 2015 [citado 16 jun 2018];(21):71-83. Disponível em: http://repositorio.ipea.gov.br/bitstream/11058/6477/1/BEPI_n21_Cria%C3%A7%C3%A3o.pdf.

[20] 20. Lotta G, Favareto A. Desafios da integração nos novos arranjos institucionais de políticas públicas no Brasil. Rev Sociol Polit. 2016;24(57):49-65. https://doi.org/10.1590/1678-987316245704

[21] 21. Arretche M. A agenda institucional. Rev Bras Cienc Soc. 2007;22(64):147-51. https://doi.org/10.1590/S0102-69092007000200011

[22] 22. Preuss LT. A gestão do Sistema Único de Saúde no Brasil e as regiões de fronteira em pauta. Rev Katalysys. 2018;21(2):324-35. https://doi.org/10.1590/1982-02592018v21n2p324

[23] 23. Silva SF. Organização de redes regionalizadas e integradas de atenção à saúde: desafios do Sistema Único de Saúde (Brasil). Cienc Saude Coletiva. 2011;16(6):2753-62. https://doi.org/10.1590/S1413-81232011000600014 10.1590/s1413-81232011000600014 21709973

[24] 24. Casanova AO, Cruz MM, Giovanella L, Alves GR, Cardoso GCP. Health care networks implementation and regional governance challenges in the Legal Amazon Region: an analysis of the QualiSUS-Rede Project. Cienc Saude Coletiva. 2017;22(4):1209-24. https://doi.org/10.1590/1413-81232017224.26562016 10.1590/1413-81232017224.26562016 28444046

[25] 25. Santos-Melo GZ, Andrade SR, Souza CRS, Erdmann AL, Meirelles BHS. Organization of the health care network in the state of Amazonas - Brazil: a documentary research. Cienc Cuid Saude. 2018;17(3):37963. https://doi.org/10.4025/cienccuidsaude.v17i3.37963

[26] 26. Fernandes JA, Figueiredo MD. Apoio institucional e cogestão: uma reflexão sobre o trabalho dos apoiadores do SUS Campinas. Physis. 2015;25;(1):287-306. https://doi.org/10.1590/S0103-73312015000100016

[27] 27. Santos-Melo GZ, Andrade SR, Ruoff AB. Health integration across international borders: an integrative review. Acta Paul Enferm. 2018;31(1):102-7. https://doi.org/10.1590/1982-0194201800015

[28] 28. Chatterji R, Saha S. Para-diplomacy: concept and the context. India Q. 2017;73(4):375-94. https://doi.org/10.1177/0974928417731638

[29] 29. Princen S, Geuijen K, Candel J, Folgerts O, Hooijer R. Establishing cross-border cooperation between professional organizations: police, fire brigades and emergency health services in Dutch border regions. Eur Urban Reg Stud. 2016;23(3):497-512. https://doi.org/10.1177/0969776414522082

[30] 30. Euzébio EF. A porosidade territorial na fronteira da Amazônia: as cidades gêmeas Tabatinga (Brasil) e Leticia (Colômbia). Cuad Geogr Rev Colomb Geogr. 2014;23(1):109-24. https://doi.org/10.15446/rcdg.v23n1.34851

[31] 31. Martínez RZ. Paradiplomacy in North America: Canadian provinces' relations with their U.S. and Mexican Counterparts. Norteamerica. 2017;12(2):87-109. https://doi.org/10.20999//nam.2017.b004

