# Supplementary material for: Integration in health: cooperation at triple international border Amazon
Source: Rev Saude Publica. 2019 Dec 23;54:05. doi: 10.11606/s1518-8787.2020054001306 (PMC9586438; doi:10.11606/s1518-8787.2020054001306)
Supplement: Translation

# Translation: Integração em saúde: cooperação na tríplice fronteira internacional amazônica

**Authors:** Giane Zupellari dos Santos-Melo, Selma Regina de Andrade, Betina Hörner Schlindwein Meirelles, Angela Maria Blatt Ortiga

**Affiliations:** I Universidade do Estado do Amazonas. Departamento de Enfermagem. Manaus, Amazonas, Brasil.; II Universidade Federal de Santa Catarina. Departamento de Pós-Graduação em Enfermagem. Florianópolis, Santa Catarina, Brasil.; III Governo do Estado de Santa Catarina. Secretaria de Estado da Saúde de Santa Catarina. Florianópolis, Santa Catarina, Brasil.

**Keywords:** Cooperação Internacional, Administração de Serviços de Saúde, Avaliação de Programas e Projetos de Saúde, Áreas de Fronteira, Saúde na Fronteira

## Abstract

**OBJETIVO:**

Descrever o alcance e as limitações das principais estratégias de cooperação em saúde, adotadas entre 2005 e 2017, no contexto da tríplice fronteira Brasil, Colômbia e Peru.

**MÉTODO:**

Estudo de caso único, explicativo, qualitativo e integrado realizado no ano de 2017, no contexto da tríplice fronteira no município de Tabatinga, Amazonas, Brasil. Como fontes de evidências foram utilizados: dados documentais, observações diretas em quatro serviços de saúde do município de Tabatinga e entrevistas com gestores da Secretaria de Estado de Saúde do Amazonas, Secretaria Municipal de Saúde de Tabatinga, Conselho Municipal de Saúde de Tabatinga e Consulado do Peru na Colômbia. Os dados foram organizados com o *software* MaxQDA12^®^.

**RESULTADOS:**

Os dados analisados demonstraram que, no período estudado, o governo federal do Brasil realizou diversos acordos de cooperação em saúde, tanto com o Peru quanto com a Colômbia, e que o governo do estado do Amazonas empreendeu estratégias para melhoria das condições de saúde da população de Tabatinga e região do Alto Solimões, as quais indiretamente alcançaram as populações dos países vizinhos, favorecendo as inter-relações entre os países da região. Quando ao governo municipal, verificou-se a existência de acordos de integração de saúde, estabelecidos informalmente, com o intuito de minimizar as adversidades da saúde local.

**CONCLUSÃO:**

As estratégias de cooperação em saúde adotadas na tríplice fronteira amazônica apresentam diferentes finalidades, benefícios e limitações. Destacam-se como benefícios a existência de acordos de cooperação em saúde entre os governos federais do Brasil, Colômbia e Peru e a presença de acordos informais de cooperação entre os governos municipais de Tabatinga (Brasil), Letícia (Colômbia) e Santa Rosa (Peru). As limitações são o desconhecimento dos gestores locais sobre os acordos de cooperação estabelecidos entre os governos federais e a falta de legitimidade dos acordos informais estabelecidos pelo governo de Tabatinga.

## INTRODUÇÃO

O Brasil mantém, na atualidade, relações diplomáticas com países da América do Sul que se estendem para as instituições de saúde. Nas últimas décadas, organizações internacionais das quais o Brasil é integrante têm proporcionado uma evolução nas tratativas de cooperação com a América do Sul, possibilitando assim um espaço de integração em saúde entre países^1^^,^^2^.

Em regiões fronteiriças, essas relações tendem a se intensificar, por serem consideradas etapas preliminares para processos de integração regional^3^. Destaca-se a cooperação entre sistemas de saúde, reconhecida como uma das principais iniciativas para favorecimento dessas aproximações^4^.

Integração em saúde em regiões de fronteiras internacionais é entendida como procedimentos de convergência, aproximação e harmonização de políticas, regulamentações e ações que resultam em facilitação de acesso às instituições comuns, com consequente permissão de consumo de serviços sociais entre os países^4^^,^^5^. Com esse entendimento, a cooperação tratada neste estudo compreende estratégias que favorecem, em maior ou menor grau, a integração em saúde nos seus diversos aspectos, desde a colaboração entre sistemas locais de saúde até a geração de conhecimento na área^4^.

Em termos globais, é enfática a defesa de que ações de saúde e distribuição de conhecimento na área não podem ficar restritas a um determinado país, por se tratar de um bem público do qual todas as pessoas e regiões deveriam se beneficiar^6^^,^^7^. Há, portanto, o reconhecimento de que a integração em saúde entre países tende a fortalecer a equidade em níveis mundiais^6^^,^^7^.

O município fronteiriço de Tabatinga, que serviu como caso neste estudo, integra a Amazônia Legal, também conhecida como “tríplice fronteira amazônica”, pois forma cidade gêmea com Letícia, capital do departamento de Amazonas, na Colômbia, e fronteira úmida com a cidade de Santa Rosa do Yavarí, departamento de Loreto, no Peru.

As condições socioeconômicas, ambientais e culturais das populações da tríplice fronteira amazônica são retratadas como precárias, devido à baixa renda populacional, ao desmatamento e ao narcotráfico existentes na região^8^. Além dessas características, os três países apresentam sistemas de saúde contrastantes, o que dificulta os processos de integração em saúde na região^9^, sendo o Brasil o único que se estrutura sob o princípio da universalidade^10^.

Estudar como são definidos os processos de integração em saúde entre os países da tríplice fronteira amazônica poderá fortalecer as discussões sobre esse tema, no sentido de se elucidar como tais mecanismos podem ser conduzidos para a melhoria das necessidades de saúde em regiões fronteiriças. Assim, o objetivo deste estudo foi de descrever o alcance e as limitações das principais estratégias de cooperação em saúde, adotadas entre 2005 a 2017, no contexto da tríplice fronteira Brasil, Colômbia e Peru.

## MÉTODO

Estudo de caso único, explicativo, qualitativo e integrado^11^, realizado no contexto da tríplice fronteira Brasil, Colômbia e Peru, tendo como caso o município de Tabatinga, Amazonas, Brasil. O município conta com 63.635 habitantes e está localizado na região Norte do país, situado à margem esquerda do rio Solimões, na microrregião do Alto Solimões, a 1.105 km da capital do estado^12^. Foi escolhido por fazer fronteira seca com o município de Letícia, extremo sul do departamento de Amazonas, na Colômbia, e com a Ilha de Santa Rosa do Yavarí, localizada no rio Maranon, província de Mariscal Ramón Castilla, departamento de Loreto, no Peru.

Para assegurar a qualidade do estudo, foram empregados testes de confiabilidade e validade^11^. O estudo utilizou como fontes de evidências dados documentais, entrevistas e observações diretas^11^, o que permitiu ampla abordagem do fenômeno estudado^13^.

A coleta dos dados documentais^11^ aconteceu no mês de março de 2017, em arquivos físicos da Secretaria Municipal de Saúde e do Conselho Municipal de Saúde de Tabatinga e em *websites* da Secretaria de Estado de Saúde do Amazonas e do Ministério da Saúde. Foram incluídos no estudo atos administrativos, decretos, leis, portarias e resoluções emitidas pelo Ministério da Saúde; atas de reuniões e resoluções emitidas pela Comissão Intergestores Bipartite do Estado do Amazonas e pela Comissão Intergestores Regional do Alto Solimões; e ofícios, resoluções e atas de reuniões emitidas pela Secretaria Municipal de Saúde e pelo Conselho Municipal de Saúde de Tabatinga, todos produzidos entre os anos de 2005 e 2017. A escolha desses documentos se deu pela autenticidade de informações sobre possíveis processos de integração em saúde em áreas fronteiriças e na tríplice fronteira. Já o recorte temporal foi considerado a partir da implantação do programa Sistema Integrado de Saúde das Fronteiras (SIS Fronteiras) no Brasil^14^. Os documentos levantados foram avaliados conforme sua estrutura e conteúdo, em relação ao tema proposto, gerando um banco de dados de 98 documentos.

As entrevistas foram do tipo “entrevistas curtas de estudo de caso”^11^ e aconteceram entre os meses de abril e novembro de 2017. Foram realizadas 12 entrevistas com gestores das secretarias de saúde estadual e municipal e do conselho municipal de saúde (Tabela 1). Os participantes foram selecionados por apresentarem poder decisório em processos de integração em saúde entre os países da tríplice fronteira. Foi entrevistado também um informante-chave do Consulado do Peru na Colômbia, que se mostrou fundamental para o entendimento do fenômeno estudado, por apresentar evidências representando um dos países estrangeiros que compõem a tríplice fronteira amazônica. Os encontros foram agendados previamente com os gestores e contavam com roteiro semiestruturado de entrevista, contendo uma questão direcionada ao entendimento do fenômeno: “É de seu conhecimento a existência de estratégias ou acordos formais ou informais que contribuam para o favorecimento da integração em saúde entre Tabatinga (Brasil), Letícia (Colômbia) e Ilha de Santa Rosa (Peru)?” As entrevistas duraram em média 60 minutos e foram conduzidas e transcritas por uma das pesquisadoras.

**Tabela 1:** Participantes do estudo de caso sobre a integração transfronteiriça em saúde na tríplice fronteira internacional amazônica, Brasil.

Gestor estadual	Secretário(a) de estado de saúde do Amazonas; Coordenador(a) da Secretaria Executiva Adjunta de Atenção Especializada do Interior da Secretaria de Estado de Saúde do Amazonas; Coordenador(a) da Comissão Intergestores Regional do Alto Solimões; Coordenador(a) da unidade de pronto atendimento de Tabatinga; Diretor(a) da Maternidade Celina Villacrez Ruiz de Tabatinga; Diretor(a) da Fundação de Vigilância em Saúde do Estado de Amazonas.
Gestor municipal	Secretário(a) municipal de saúde de Tabatinga; Presidente do Conselho Municipal de Saúde de Tabatinga; Coordenador(a) de atenção básica do município de Tabatinga; Coordenador(a) da unidade básica de saúde da família Dídimo Pires de Oliveira; Coordenador(a) da unidade básica de saúde da família Santa Rosa.
Informante-chave	Cônsul do Peru na Colômbia.

As observações diretas^11^ aconteceram nos meses de novembro e dezembro de 2017, gerando um total de 34 horas de observação. Foram incluídas no estudo duas unidades básicas de saúde da família (UBSF), selecionadas como pontos de observação por estarem situadas nas proximidades das linhas das fronteira internacionais. Essas duas UBSF são administradas pela Secretaria Municipal de Saúde e estão situadas nos bairros de São Francisco e Santa Rosa.

Outros serviços observados foram uma unidade de pronto atendimento e uma maternidade estadual, escolhidas como ponto de observação por serem referências para atendimento de urgência e emergência e partos de baixo risco na região do Alto Solimões, além de prestarem atendimento de saúde a estrangeiros. As duas unidades são administradas pela secretaria estadual, porém situadas no bairro Vila Paraíso, em Tabatinga.

Para direcionamento das observações, foi utilizado um roteiro constando: local, data e horário de início e término das observações; eventos observados; e ações ou situações que demonstrassem a existência ou necessidade de existência de acordos de integração em saúde entre os países da tríplice fronteira.

Os dados foram organizados com auxílio de *software* MAXQDA12^®^, que permitiu a criação de códigos e subcódigos^11^. A confrontação das três fontes de evidências possibilitou a triangulação de dados relativos à existência de acordos informais concernentes aos resultados apresentados (Figura 1).

**Figura:** Triangulação de dados evidenciando a existência de acordos informais de integração em saúde na tríplice fronteira internacional amazônica Brasil, Colômbia e Peru.

Fonte: MaxQDA12^®^, 2018.

Obs: observação

As diretrizes éticas do Conselho Nacional de Saúde foram respeitadas em todas as fases do estudo, que recebeu aprovação do Comitê de Ética e Pesquisa da Universidade Federal de Santa Catarina, com Parecer nº 2.047.137.

## RESULTADOS E DISCUSSÂO

Evidenciou-se que no período estudado foram realizados diversos acordos de cooperação em saúde entre os governos do Brasil e Colômbia e Brasil e Peru, conforme pode ser verificado na Tabela 2. Apesar de esses acordos permanecerem em vigor, verificou-se que os participantes deste estudo ou ignoram sua existência ou não os reconhecem como possíveis ferramentas para o favorecimento da integração em saúde na região estudada.

**Tabela 2:** Acordos de integração em saúde entre Brasil e Colômbia ou Brasil e Peru, de 2005 a 2017.

Ano*	Países	Objetivo do acordo	Vigência
2006	Brasil e Peru	Fortalecimento da regulamentação e fiscalização em saúde pública no processo de descentralização dos ministérios da saúde do Brasil e do Peru.	Em vigor
2006	Brasil e Peru	Fortalecimento institucional das assessorias internacionais dos ministérios da saúde do Brasil e do Peru.	Em vigor
2007	Brasil e Colômbia	Apoio técnico para a implementação de bancos de leite humano na Colômbia.	Em vigor
2007	Brasil e Colômbia	Fortalecimento institucional das assessorias internacionais dos ministérios da saúde do Brasil e da Colômbia.	Em vigor
2009	Brasil e Colômbia	Fortalecimento no diagnóstico molecular e tipificação das espécies de *Leishmania*, sua georreferenciação e análise espacial.	Em vigor
2010	Brasil e Peru	Estabelecimento de uma zona de integração fronteiriça Brasil-Peru para a criação do subgrupo de trabalho sobre saúde na fronteira.	Em vigor
2012	Brasil e Colômbia	Pesquisa e desenvolvimento para a fabricação e controle da qualidade de produtos biológicos na Colômbia.	Em vigor
2013	Brasil e Peru	Estabelecimento de quadro complementar para o desenvolvimento de um novo programa de cooperação entre o Ministério da Saúde do Brasil e órgãos vinculados e o Ministério da Saúde do Peru.	Em vigor

* Ano de promulgação do acordo.

Acordo formal que envolva a tríplice fronteira? Eu desconheço. […] Só se forem internos na própria região, […] mas para abranger os municípios dos países vizinhos, não – ou seja, essas questões internacionais. (Gestor 8)

Eu soube que foram assinados alguns acordos bilaterais, mas eu sei que na realidade, aqui, esses acordos nunca funcionaram. […] Eu sei que eles existem, mas não funcionam. (Gestor 6)

Os acordos celebrados entre os governos federais do Brasil, Colômbia e Peru, seguem a ordem global, que cada vez mais compreende que a cooperação internacional é uma iniciativa esperada entre os países, por acreditar-se que o intercâmbio de conhecimentos e experiências pode equiparar nações^15^. Porém, para que essa cooperação apresente resultados efetivos, é necessária a criação de normas, mobilização de recursos e orientação das várias partes envolvidas^16^, que em regiões fronteiras são os residentes, gestores e profissionais de saúde^10^.

Este estudo levantou dados sobre duas estratégias, estabelecidas pelo governo federal, com capacidade de fomentar a integração em saúde na região estudada. A primeira delas foi o SIS Fronteiras, criado em 2005 com o objetivo de avaliar as condições de saúde dos municípios fronteiriços e promover a integração de ações e serviços de saúde. O município de Tabatinga aderiu ao programa em 2006, porém os dados analisados demonstraram que, na região da tríplice fronteira amazônica, poucos avanços foram favorecidos por ele.

A questão de integração, eu não sei como o SIS Fronteiras estava pretendendo fazer isso, porque aqui na região, não aconteceu nada em relação a essa proposta. (Gestor 6)

A segunda estratégia foi o Plano de Desenvolvimento e Integração das Faixas de Fronteira (PDIFF), que tem por objetivo promover o desenvolvimento da faixa de fronteira por meio de estruturação física, social e produtiva. Essa estratégia dialogou com gestores da tríplice fronteira Brasil, Colômbia e Peru. No entanto, esse diálogo não aumentou as expectativas e a credibilidade em possíveis mudanças nas políticas dirigidas à integração em saúde para região.

Eu pude participar de uma dessas reuniões em Brasília […] Eu falei sobre as dificuldades de saúde, […] mas é aquela coisa: para a reunião eles se colocaram à disposição, mas eu voltei de lá já tem um tempo e ninguém mais se manifestou sobre isso. (Gestor 6)

Apesar de essas duas estratégias apresentarem em seus objetivos o favorecimento de integração em saúde em regiões fronteiriças, os participantes não reconhecem sua efetividade na região estudada. No caso do SIS Fronteiras, esse fato pode estar relacionado a condição de que, em municípios longínquos e de pequeno porte, como o estudado, a implantação do programa se deu de forma verticalizada, com um exíguo debate entre os órgãos ministeriais, estaduais e municipais, levando ao desconhecimento e más interpretações do programa e seus resultados^17^. Para esses municípios, a concepção do SIS Fronteiras foi vinculada aos repasses financeiros para atenção à saúde de estrangeiros, sendo pouco associada às cooperações transfronteiriças.

Quanto ao governo do estado do Amazonas, pode-se verificar que empreendeu estratégias para melhoria das condições de saúde da população local, que indiretamente alcançaram os residentes dos países vizinhos que buscam atendimento de saúde em Tabatinga, consequentemente beneficiando a inter-relação entre os três países. Entretanto, observou-se que essas estratégias não foram formuladas especificamente para o favorecimento da integração em saúde na tríplice fronteira, tampouco para a normatização da atenção à saúde de residentes nos países limítrofes ao Brasil, fazendo com que a prática do atendimento de saúde ao estrangeiro permaneça uma discricionariedade do gestor local.

Ao reconhecer as peculiaridades das regiões fronteiriças que compõem o seu território, o governo estadual empreende tais estratégias, mediante políticas verticalizadas do governo federal, para dar respostas às necessidades de saúde da população brasileira daquela região. Contudo, mesmo admitindo a existência de dificuldades relativas à atenção à saúde de estrangeiros, esse nível governamental não promove ações diretas que favoreçam a integração de saúde, pois lhe falta autonomia político-administrativa para estabelecer acordos internacionais com os países limítrofes. Entes não centrais como estados e municípios podem estabelecer acordos de cooperação formais com governos internacionais, desde que estes tenham apreciação do poder central e chancela do Senado Federal, porém tais acordos apresentam fragilidade legal, por não existirem instrumentos jurídicos que os amparem^18^.

Nesse contexto, a baixa autonomia dos governos locais resulta em dificuldades na coalizão de medidas para melhoria de saúde das populações fronteiriças. Independentemente de a Constituição Federal elevar os estados a entes federativos, isso não legitima que eles estabeleçam de forma autônoma execuções com organismos internacionais, ficando assim dependentes de diretrizes propostas pelo governo federal^19^. Essa dependência é gerada pelo modelo verticalizado de implementação de políticas sociais, incluindo as de saúde, do governo brasileiro^20^, uma vez que mecanismos institucionais como a concentração de recursos tributários na União limitam a autonomia decisória dos governos locais^21^. Assim, para que políticas desta natureza possam ser efetivas, devem ser planejadas horizontalmente, respeitando as diretrizes de intersetorialidade, relações entre governos federal, estaduais e municipais e as dimensões políticas e territoriais^20^.

No caso de regiões fronteiriças, o pacto pela saúde trouxe avanços ao contemplar os territórios fronteiriços em seu projeto de regionalização. Além de assegurar o cumprimento dos princípios de universalidade, equidade e integralidade das ações e serviços de saúde, essa determinação ampliou a capacidade operacional dos municípios. No entanto, a implementação desta norma depende da possibilidade de esses municípios exercerem sua autonomia na gestão de saúde^22^.

Dentre as estratégias implementadas pelo governo do estado do Amazonas, três apresentaram destaque para melhoria das condições de saúde da tríplice fronteira. Uma delas foi o Projeto de Desenvolvimento Regional do Estado do Amazonas (Proderam), implantado em Tabatinga em 2003, com o objetivo melhorar a qualidade de vida da população local por meio de acesso à saúde, melhoria dos sistemas de saneamento básico e aumento da oferta de empregos e renda. Outra estratégia foi o Projeto de Formação e Melhoria da Qualidade da Rede de Saúde (QualiSUS-Rede), que objetivou contribuir para a organização de redes regionalizadas de atenção à saúde no Brasil. Esse programa avançou na região estudada a partir de 2012.

Essas duas estratégias foram essenciais para a criação da Rede de Atenção à Saúde do Alto Solimões (RAS-AS), da qual Tabatinga é município-polo. Com a experiência do Proderam, a região do Alto Solimões foi contemplada como uma das 15 regiões de saúde a participarem do QualiSUS-Rede na região amazônica. A RAS-AS proporcionou a Tabatinga a infraestrutura de atenção à urgência e emergência e à saúde da mulher e da criança. Essas estratégias mudaram as características da região, inclusive na atenção à saúde de estrangeiros, que passaram a contar com esses serviços.

*Normalmente vienes por situaciones de emergencia […] los ciudadanos peruanos terminen siendo atendidos en Tabatinga […] en la UPA.* (Gestor 11)

O Pacto pela Saúde, na dimensão do Pacto de Gestão, determina o desenho de redes regionalizadas de atenção à saúde no Sistema Único de Saúde (SUS). Essa proposta evoluiu para as redes de atenção à saúde, que são na atualidade uma das principais propostas de funcionamento dos serviços de saúde^23^. A efetivação da RAS-AS foi uma estratégia, na qual se observou uma leve alteração na inter-relação entre os governos federal e estadual, pois as condições de saúde e geografia da região da tríplice fronteira foram consideradas prioridade para implantação de uma rede de atenção à saúde naquela região^24^^,^^25^.

A RAS-AS é entendida pela maioria dos participantes como passível de diminuir as iniquidades socias da região. Entretanto, alguns alegam que, apesar de melhorar a resolutividade do setor de saúde, ela não contempla a magnitude da realidade local. Para eles, as respostas são impostas por instruções normativas que nem sempre correspondem às necessidades sentidas pela população fronteiriça.

A saúde tenta dar respostas em cima do geral, através da assistência de atenção primária, da urgência e emergência, internações, parto e nascimento, […] mas nós sabemos que isso não alcança as necessidades deles. (Gestor 7)

Assim, a fim de reconhecer as reais necessidades da região, o governo estadual desenvolveu a estratégia de manter apoiadores institucionais na RAS-AS. Sua função é observar a dinâmica de saúde e propor ações que possam minimizar as desigualdades socias encontradas.

Nossa visão hoje é por região. O estado vai intervir na tríplice fronteira através dos apoiadores institucionais e do diagnóstico situacional da região. […] Esse apoiador institucional vai monitorar os municípios da região para entender e dar um melhor trato às especificidades locais. (Gestor 7)

A concepção de apoio institucional no setor de saúde é um mecanismo de gestão baseado no método Paideia, que transforma a maneira de realizar gestão em saúde. Pela cogestão entre gestores, trabalhadores e usuários, esse método é capaz de expandir a análise reflexiva do coletivo. Assim, o apoiador institucional é considerado um recurso para construção de processos de mudanças em grupos e organizações de saúde^26^.

Quanto às estratégias estabelecidas pelo governo municipal para favorecer a integração, observou-se a ocorrência de intensa movimentação de acordos de cooperação em saúde realizados informalmente entre Tabatinga e os dois municípios dos países vizinhos. Esses acordos são em sua maioria celebrados entre os municípios colombiano e brasileiro e utilizados tanto para troca de informações quanto para utilização de bens e serviços em saúde. A fala de um dos participantes deixa claro que são comuns na região:

Hoje, nós trabalhamos a integração, mesmo que seja de forma não formal, mas existe essa integração, […] que aqui é meio que automática, porque nós temos que nos adaptar a esse fenômeno que é a fronteira. E como fazemos isso? Fazendo parcerias. (Gestor 6)

Esses tipos de acordo, apesar de não reconhecidos legalmente^19^, são comuns entre municípios de fronteiras internacionais, tanto no Brasil como em outros países^27^. Na atualidade, esse fenômeno, descrito como paradiplomacia, vem ganhando destaque nas discussões das relações internacionais e redefinindo o papel das relações exteriores do mundo, principalmente nas Américas, Europa Ocidental e Ásia^28^.

A paradiplomacia pode ser definida como a participação de entes federativos ou regionais em assuntos internacionais, como execução de tratados com Estados estrangeiros, participação em redes internacionais de cooperação regional e atuação em políticas externas, sem o amparo dos governos centrais^29^.

Em regiões fronteiriças, o fenômeno da paradiplomacia tende a se intensificar, pois as necessidades mútuas das regiões, normalmente marcadas por desequilíbrios socioeconômicos e ambientais, assim como as características geográficas, fazem com que governos e instituições não governamentais se unam no intuito de minimizar as iniquidades regionais^27^. Com isso, esse tipo de cooperação transfronteiriça permite que governos locais com recursos escassos e baixa presença dos estados-nações encontrem soluções objetivas, rápidas e criativas para os problemas locorregionais^29^.

A paradiplomacia é utilizada pelos gestores de saúde da tríplice fronteira amazônica como uma estratégia para contornar as adversidades próprias da região, principalmente as relacionadas à logística, que é dificultada pela elevada distância entre o município e a capital do estado, e para minimizar a baixa resolutividade dos serviços de saúde locais. Participantes do estudo declaram que essa ferramenta tem sido fundamental para o desenvolvimento de ações de saúde da região.

A usina do abastecimento de oxigênio daqui não dá conta de suprir as nossas necessidades, […] então quem me abastece é Letícia. […] Isso por acordos informais. (Gestor 3)

Se uma hora eles precisarem de alguma coisa, e a gente tiver, a gente vai ajudar, e se nós precisarmos de alguma coisa, se eles puderem, também sempre ajudam. Mas isso depende de contatos. […] Tudo é acordo de cavalheiros, ou seja, de boa vizinhança. (Gestor 6)

Pelas leis nacionais, o município brasileiro da tríplice fronteira amazônica pode celebrar tais acordos, desde que estes sejam chancelados pelo governo central^18^^,^^19^. Já o município colombiano de Letícia é amparado pela Constituição Federal da Colômbia, que permite que municípios fronteiriços estabeleçam acordos binacionais para o desenvolvimento econômico e articulações com os países vizinhos, desde que sejam voltados para a melhoria das condições de vida e inclusão social de comunidades isoladas. Diante disso, a embaixada da Colômbia em Tabatinga permite o estabelecimento de acordos de cooperação entre os dois municípios em temas relacionados à educação, segurança pública, transporte e saúde^30^.

As diferenças nas tratativas jurídicas relacionadas a este tópico nos dois países causam situações de insegurança para os gestores do lado brasileiro, que reconhecem que os acordos existentes, na atualidade, não apresentam validade jurídica e declaram desconforto em promovê-los. No entanto, afirmam que eles constituem uma alternativa local de suprir a falta de ações que favoreçam a integração em saúde na região por instâncias superiores.

Isso tudo é uma coisa que me deixa muito inquieto, porque não existe algo formal em uma esfera superior. (Gestor 3)

Apesar de as normativas nacionais indicarem que acordos dessa natureza não são oficiais, o governo federal brasileiro segue diretrizes de aceitabilidade da paradiplomacia, o que confere certa autonomia para municípios fronteiriços brasileiros realizarem essas transações. A prerrogativa de aceitabilidade da paradiplomacia em regiões fronteiriças já é reconhecida em países como Canadá, México, Alemanha, Argentina, África do Sul e Estados Unidos, por serem consideradas benéficas, dada a diversidade política que elas proporcionam^19^^,^^20^^,^^31^.

## CONCLUSÃO

Por serem territórios normalmente isolados e com características socioeconômicas peculiares, as fronteiras internacionais são reconhecidas como territórios favoráveis ao desenvolvimento de processos de integração políticos, econômicos e culturais que tendem a minimizar as desigualdades existentes nesses locais. Este estudo buscou elucidar a ocorrência de estratégias de cooperação em saúde na região da tríplice fronteira Brasil, Colômbia e Peru. Foi observado que tais estratégias existem, porém apresentam diferentes finalidades, dependendo das políticas envolvidas e do nível governamental que as estabelecem. Os acordos firmados apresentam pouca efetividade na região, e o governo do estado do Amazonas não promove estratégias específicas para o favorecimento da integração em saúde.

Com isso, ocorre um distanciamento entre o município e os demais níveis governamentais, resultando na ocorrência rotineira de acordos de cooperação informais entre Tabatinga, Letícia e Ilha de Santa Rosa. Esses acordos representam uma alternativa para minimização das iniquidades de saúde e poderiam servir como precedentes para futuras propostas de integração entre instâncias superiores dos governos do Brasil, Colômbia e Peru. Em concordância com os termos de saúde global, o envolvimento dos governos federal e estadual em negociações dessa natureza pode favorecer a melhoria das condições na região, tornando o acesso a bens e serviços de saúde menos excludente e mais igualitário.
